# Features of the Generalized Dynamics of Quasiparticles in Graphene

**DOI:** 10.1186/s11671-017-1940-0

**Published:** 2017-03-09

**Authors:** Anatol D. Suprun, Liudmyla V. Shmeleva

**Affiliations:** Department of Theoretical Physics, Faculty of Physics, Taras Shevchenko National University of Kyiv, Volodymyrska Street, 64/13, Kyiv, 01601 Ukraine

**Keywords:** Graphene, Dispersion law, Hamiltonian, Lagrangian, Velocity, Effective mass, Momentum

## Abstract

The general dynamic properties of the electron, as quasiparticle in conduction band of graphene, were analyzed. It is shown that in graphene, these properties essentially differ from similar base properties for crystals with a simple lattice, despite insignificant, on the first sight, difference of dispersion law *ε*(**p**). Primarily, crystals with an elementary cell of arbitrary complexity of structure were considered. The obtained general relations were applied further to graphene. Herewith two-dimensional lattice of graphene has been considered as consisting of elementary cells with two atoms. Typically, graphene is considered as crystals consisting of two simple nested sublattices. It has been shown that both considerations lead to the analogous basic results. On the basis of obtained wave Hamiltonian, all the dynamic characteristics of the injected electron, considered as a quasiparticle, were found: speed, tensor of effective dynamic mass, and wave Lagrangian. Also, for some physically actual situations, the dynamic characteristics of an alternative description have been found: a mechanical momentum **p**_*m*_, mechanical Hamiltonian, and mechanical Lagrangian. For these situations, a generalized Louis de Broglie relationship between mechanical **p**_*m*_ and wave **p** momenta was found also.

## Background

Due to the growing interest in physical, particularly electrically conductive properties of graphene, the analysis of general dynamic properties of the electron, as quasiparticle in the band of conductivity or excitons (bound electron-hole pair) is particularly relevant. Such dynamic properties are associated with the study of charge and energy transfer processes. General theoretical concepts of these processes are developed intensively as in graphene [1–8] so in other physical systems [9–16]. The theoretical description of charge or energy transfer processes can also be used for research in sphere of transmission of information signals [17] and be the basis for further development in areas such as superconductivity [18] and superfluidity [19]. Such an analysis can be relevant for other environments [20–22], non-crystalline. Everything leads to the need for a general analysis of the dynamic properties of quasiparticles in crystals in general and for graphene, in particular.

Analysis of the general dynamic properties of an electron in the conduction band was carried out (as before [23–27]), on the base of one of the main characteristics of excited states of condensed matter: on the basis of the dispersion law.

Typically, graphene is considered as flat carbon monatomic crystals consisting of two simple nested sublattices [1–5]. However, such a consideration differs from the traditional one. This last is based on the concept of a complex unit cell with a few atoms.

The purpose of the article is to show that the traditional consideration leads to the already known as basic results for graphene, to fulfill the analysis of general dynamic properties of the electron (considering as a quasiparticle) in the conduction band of graphene and to compare obtained results with the analogous basic characteristics for the simple lattices.

It is shown that all the dynamic characteristics are significantly different from the standard ones for crystals with a simple lattice [24–27].)

## Methods

### The Basic System of Equations for Quasiparticles in the Crystals With a Complex Unit Cell

It is known [28] that in the case of crystals with a complex unit cell (several atoms in one cell), the Hamilton functional for the one-electron excitations without account of lattice response on the excitation [27] is defined by the equation [24]:

$$ E\left(\left\{ a\right\}\right)=\frac{1}{2}\left\{{\displaystyle \sum_{{\boldsymbol{n}}_{\alpha}{\boldsymbol{m}}_{\beta}}{}^{/} w_{{\boldsymbol{n}}_{\alpha}{\boldsymbol{m}}_{\beta}}}+{\displaystyle \sum_{{\boldsymbol{n}}_{\alpha}}2{D}_{{\boldsymbol{n}}_{\alpha}}{\left|{a}_{{\boldsymbol{n}}_{\alpha}}\right|}^2}+{\displaystyle \sum_{{\boldsymbol{n}}_{\alpha}{\boldsymbol{m}}_{\beta}}{}^{/} M_{{\boldsymbol{n}}_{\alpha}{\boldsymbol{m}}_{\beta}}\left({a}_{{\boldsymbol{n}}_{\alpha}}^{*}{a}_{{\boldsymbol{m}}_{\beta}}+{a}_{{\boldsymbol{m}}_{\beta}}^{*}{a}_{{\boldsymbol{n}}_{\alpha}}\right)}\right\}. $$

Vectors ***n***_*α*_, ***m***_*β*_ are defined by the relations:

1 $$ {\boldsymbol{n}}_{\alpha}=\boldsymbol{n}+{\boldsymbol{r}}_{\alpha} $$

where ***n*** = *n*_*i*_ ***b***_*i*_ is standard lattice vector. Index *i* accepts from one to three values, depending on the dimension of the crystal (for one-dimensional crystal (polymers) *i* = 1 for two-dimensional crystals (graphene) *i* = {1, 2}; for three-dimensional crystals *i* = {1, 2, 3}); *n*_*i*_ are integers corresponding to the number of unit cells along the crystallographic direction *i*: *n*_*i*_ = 0, ± 1, ± 2, ….Lattice vectors ***b***_*i*_ are defined conventionally: ***b***_*i*_ = *b*_*i ξ*_ ***e***_*ξ*_, where *b*_*i ξ*_ is vectors projection of ***b***_*i*_ on to unit vectors ***e***_*ξ*_ = (***e***_*x*_, ***e***_*y*_, ***e***_*z*_) of Cartesian axes. Indices *α*, *β* take integer values from 0 to *S* − 1, where *S* is the number of atoms per unit cell. Value *α* = 0 corresponds to the main unit cell atom, which defines the cell itself. It is assumed that ***r***_0_ = 0. For values *α* ≠ 0 the vectors ***r***_*α*_ are identified by the obvious relations: ***r***_*α*_ = ***r***_*αξ*_ ***e***_*ξ*_, where cp are coordinates of atom *α* ≠ 0.

Using the procedure of Hamiltonian dynamic minimization [10, 26], in the approximation of an ideal lattice and with account of the representation (1), the following system of equations can be obtained:

2 $$ i\hslash \frac{\partial {a}_{{\mathbf{n}}_{\alpha}}}{\partial t}={D}_{\alpha}{a}_{{\boldsymbol{n}}_{\alpha}}+{\displaystyle \sum_{\beta \left(\ne \alpha \right)}{M}_{{\boldsymbol{r}}_{\beta}-{\boldsymbol{r}}_{\alpha}}\kern0.5em {a}_{{\boldsymbol{n}}_{\beta}}}+{\displaystyle \sum_{\boldsymbol{m}\left(\ne \boldsymbol{n}\right)}{M}_{\boldsymbol{m}-\boldsymbol{n}}\kern0.5em {a}_{{\boldsymbol{m}}_{\alpha}}}+{\displaystyle \sum_{\boldsymbol{m}\left(\ne \boldsymbol{n}\right)}{\displaystyle \sum_{\beta \left(\ne \alpha \right)}{M}_{\boldsymbol{m}-\boldsymbol{n}+{\boldsymbol{r}}_{\beta}-{\boldsymbol{r}}_{\alpha}}\kern0.5em {a}_{{\boldsymbol{m}}_{\beta}}}} $$

the solution of which has the following form:

3 $$ {a}_{{\boldsymbol{n}}_{\alpha}}={A}_{\alpha}{e}^{i\;\left[\left(\boldsymbol{k}\cdot \boldsymbol{n}\right)-\omega t\right]} $$

Here, in contrast to the simple lattice (one atom per unit cell), the coefficients *A*_*α*_ are complex values. Then (2) takes the form of an algebraic system for the coefficients *A*_*α*_ and ${A}_{\alpha}^{*}$ determination:

4 $$ \begin{array}{c}\left({D}_{\alpha}+{\displaystyle \sum_{\boldsymbol{n}\left(\ne \mathbf{0}\right)}{M}_{\boldsymbol{n}}{e}^{i\;\left(\boldsymbol{k}\cdot \boldsymbol{n}\right)}}-\mathit{\hslash \omega}\right){A}_{\alpha}+{\displaystyle \sum_{\beta \left(\ne \alpha \right)}{M}_{{\boldsymbol{r}}_{\beta}-{\boldsymbol{r}}_{\alpha}}{A}_{\beta}}+{\displaystyle \sum_{\beta \left(\ne \alpha \right)}{A}_{\beta}{\displaystyle \sum_{\boldsymbol{n}\left(\ne \mathbf{0}\right)}{M}_{\boldsymbol{n}+{\boldsymbol{r}}_{\beta}-{\boldsymbol{r}}_{\alpha}}{e}^{i\;\left(\boldsymbol{k}\cdot \boldsymbol{n}\right)}}}=0\kern0.5em ;\\ {}\left({D}_{\alpha}+{\displaystyle \sum_{\boldsymbol{n}\left(\ne \mathbf{0}\right)}{M}_{\boldsymbol{n}}{e}^{- i\;\left(\boldsymbol{k}\cdot \boldsymbol{n}\right)}}-\mathit{\hslash \omega}\right){A}_{\alpha}^{*}+{\displaystyle \sum_{\beta \left(\ne \alpha \right)}{M}_{{\boldsymbol{r}}_{\beta}-{\boldsymbol{r}}_{\alpha}}{A}_{\beta}^{*}}+{\displaystyle \sum_{\beta \left(\ne \alpha \right)}{A}_{\beta}^{*}{\displaystyle \sum_{\boldsymbol{n}\left(\ne \mathbf{0}\right)}{M}_{\boldsymbol{n}+{\boldsymbol{r}}_{\beta}-{\boldsymbol{r}}_{\alpha}}{e}^{- i\;\left(\boldsymbol{k}\cdot \boldsymbol{n}\right)}}}=0\kern0.5em \end{array} $$

Non-trivial solution of the system (4) with respect to the *A*_*α*_, ${A}_{\alpha}^{*}$ coefficients is defined by the condition of its consistency. However, this condition can be implemented only under concretization of the crystal lattice.

## Results and Discussion

### Graphene as a Flat Monolayer Crystal Containing two Atoms per Unit Cell

In this subsection, we show that graphene consideration as a crystal with a complex unit cell leads to the basic results, analogous to the consideration of two nested interacting crystals with simple unit cells. In particular, it concerns such a main characteristic as the electron energy in the conduction band (dispersion *ε*(**p**)). Here, we will proceed from the general system (4) for any crystals.

**Fig. 1:**
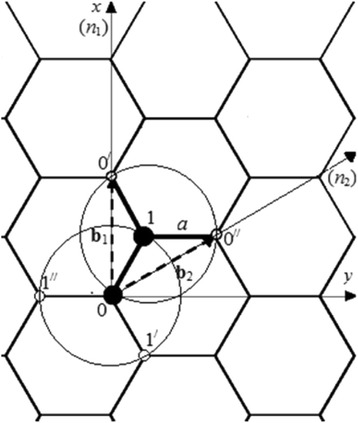
The crystal lattice of graphene and its elementary cell. Basic distance between atoms is denoted by *a*. The vectors ***b***_1_, ***b***_2_ determines the crystallographic directions. The unit cell contains two atoms which are marked by numerals “*0*” (basic atom) and “*1*” (an extra atom). Numerals “*0* ^*/*^”, “*0* ^*/*/^” denote the atoms of neighboring unit cells equivalent to atom “0”. Numerals “*1* ^*/*^”, “*1* ^*//*^” denote the atoms of neighboring unit cells equivalent to atom “*1*”. Two circles with centers in atoms “*0*” and “*1*” shows the first two coordinative “*spheres*,” which limit the consideration by the approximation of the nearest neighbors

Fig. 1 shows a fragment of the graphene lattice and its unit cell with two atoms. According to the definition (1), a two-dimensional lattice vector ***n*** = *n*_*i*_ ***b***_*i*_ is defined by the equation: ***n*** = *n*_1_ ***b***_1_ + *n*_2_ ***b***_2_. The unit cell is shown in detail in Fig. 1 and corresponds to the values *n*_1_ = *n*_2_ = 0. The following can be established by its geometry: ***b***_1_ = ***e***_*x*_ *b*, ${\boldsymbol{b}}_2={\boldsymbol{e}}_x\frac{1}{2} b+{\boldsymbol{e}}_y\frac{3}{2} a$, where $b= a\sqrt{3}$. Then the following can be obtained for the vector ***n***: $\boldsymbol{n}={\boldsymbol{e}}_x\left({n}_1+\frac{1}{2}{n}_2\right) b+{\boldsymbol{e}}_y\frac{3}{2}{n}_2\kern0.1em  a$.

Analogically, in accordance with (1), for the vector ***r***_*α*_ = *r*_*αξ*_ ***e***_*ξ*_, which is reduced to a form: ***r***_1_ = *r*_1*x*_ ***e***_*x*_ + *r*_1*y*_ ***e***_*y*_, also can be derived from Fig. 1: ${r}_{1 x}=\frac{b}{2}=\frac{\sqrt{3}}{2} a$; ${r}_{1 y}=\frac{a}{2}$. Then it is obvious that ${\boldsymbol{r}}_1=\frac{b}{2}{\boldsymbol{e}}_x+\frac{a}{2}{\boldsymbol{e}}_y$.

Considering further that ***k*** = ***e***_*x*_ *k*_*x*_ + ***e***_*y*_ *k*_*y*_, the scalar product (***k*** ⋅ ***n***) of the factors *e* ^± *i* (***k*** ⋅ ***n***)^ in determining of the system (4) is reduced to the form: (***k*** ⋅ ***n***) = *n*_1_ *p*_1_ + *n*_2_ *p*_2_. The dimensionless momenta *p*_1_ and *p*_2_ have the form of:

$$ {p}_1=\left(\boldsymbol{k}\cdot {\boldsymbol{b}}_1\right)={k}_x b\equiv {p}_x;\kern1em {p}_2=\left(\boldsymbol{k}\cdot {\boldsymbol{b}}_2\right)=\frac{1}{2}{p}_x+\frac{3}{2}{p}_y $$

where *p*_*y*_≡*k*_*y*_ *a*. The subsequent consideration is more convenient to execute for *p*_1_, *p*_2_ momenta. To the *p*_*x*_, *p*_*y*_ momenta we will return below.

Further, in the Eq. (4) were taken into account all properties of the crystal lattice. Also the representation ${e}^{\pm i\kern0.1em {p}_{1,2}}= \cos \left({p}_{1,2}\right)\pm i \sin \left({p}_{1,2}\right)$ was taken into account. Then, in the nearest-neighbor approximation (within the first two coordination “spheres”, shown in Fig. 1), the commonly used system of equations written in matrix form [1–5] was obtained:

5 $$ {M}_a\left\{1+ \cos \left({p}_1\right)+ \cos \left({p}_2\right)\right\}{\widehat{\varSigma}}_1\boldsymbol{A}+{M}_a\left\{ \sin \left({p}_1\right)+ \sin \left({p}_2\right)\right\}{\widehat{\varSigma}}_2\boldsymbol{A}+\widehat{D}\boldsymbol{A}=\hslash \omega \widehat{I}\boldsymbol{A} $$

where

$$ \begin{array}{l}\mathbf{A}=\left(\begin{array}{c}\hfill {A}_0\hfill \\ {}\hfill {A}_1\hfill \\ {}\hfill {A}_0^{*}\hfill \\ {}\hfill {A}_1^{*}\hfill \end{array}\right);\kern1em {\widehat{\varSigma}}_1=\left(\begin{array}{cccc}\hfill 0\hfill & \hfill 1\hfill & \hfill 0\hfill & \hfill 0\hfill \\ {}\hfill 1\hfill & \hfill 0\hfill & \hfill 0\hfill & \hfill 0\hfill \\ {}\hfill 0\hfill & \hfill 0\hfill & \hfill 0\hfill & \hfill 1\hfill \\ {}\hfill 0\hfill & \hfill 0\hfill & \hfill 1\hfill & \hfill 0\hfill \end{array}\right)\equiv \left(\begin{array}{cc}\hfill {\widehat{\sigma}}_x\hfill & \hfill \widehat{0}\hfill \\ {}\hfill \widehat{0}\hfill & \hfill {\widehat{\sigma}}_x\hfill \end{array}\right);\kern1em {\widehat{\varSigma}}_2=\left(\begin{array}{cccc}\hfill 0\hfill & \hfill - i\hfill & \hfill 0\hfill & \hfill 0\hfill \\ {}\hfill i\hfill & \hfill 0\hfill & \hfill 0\hfill & \hfill 0\hfill \\ {}\hfill 0\hfill & \hfill 0\hfill & \hfill 0\hfill & \hfill i\hfill \\ {}\hfill 0\hfill & \hfill 0\hfill & \hfill - i\hfill & \hfill 0\hfill \end{array}\right)\equiv \left(\begin{array}{cc}\hfill {\widehat{\sigma}}_y\hfill & \hfill \widehat{0}\hfill \\ {}\hfill \widehat{0}\hfill & \hfill -{\widehat{\sigma}}_y\hfill \end{array}\right)\\ {}\kern6em \widehat{D}=\left(\begin{array}{cccc}\hfill {D}_0\hfill & \hfill 0\hfill & \hfill 0\hfill & \hfill 0\hfill \\ {}\hfill 0\hfill & \hfill {D}_1\hfill & \hfill 0\hfill & \hfill 0\hfill \\ {}\hfill 0\hfill & \hfill 0\hfill & \hfill {D}_0\hfill & \hfill 0\hfill \\ {}\hfill 0\hfill & \hfill 0\hfill & \hfill 0\hfill & \hfill {D}_1\hfill \end{array}\right);\kern2em \widehat{I}=\left(\begin{array}{cccc}\hfill 1\hfill & \hfill 0\hfill & \hfill 0\hfill & \hfill 0\hfill \\ {}\hfill 0\hfill & \hfill 1\hfill & \hfill 0\hfill & \hfill 0\hfill \\ {}\hfill 0\hfill & \hfill 0\hfill & \hfill 1\hfill & \hfill 0\hfill \\ {}\hfill 0\hfill & \hfill 0\hfill & \hfill 0\hfill & \hfill 1\hfill \end{array}\right)\end{array} $$

The matrices ${\widehat{\varSigma}}_1$, ${\widehat{\varSigma}}_2$ definition also shows the presentation by means of the Pauli matrices ${\widehat{\sigma}}_x$, ${\widehat{\sigma}}_y$, which is often used.

Finally, to determine their eigenvalue *ℏω*, the matrix Eq. (5) must be represented in the form of a system of four equations for *A*_0_, *A*_1_, ${A}_0^{*}$, ${A}_1^{*}$ coefficients. Since the resulting equations are homogeneous, the eigenvalues are determined by the conditions of this system consistency:

$$ \left({D}_0-\mathit{\hslash \omega}\right)\left({D}_1-\mathit{\hslash \omega}\right)={M}_a^2\left\{1+2\left[1+ \cos \left({p}_1\right)+ \cos \left({p}_2\right)+ \cos \left({p}_2-{p}_1\right)\right]\right\} $$.

In ideal crystal conditions (infinite and defect-free), the following condition is implemented *D*_0_ = *D*_1_ = *D*. In this approximation, the eigenvalues are of the following form:

$$ \hslash {\omega}_{\pm }= D\pm {M}_a\sqrt{1+2\left(1+ \cos \left({p}_1\right)+ \cos \left({p}_2\right)+ \cos \left({p}_2-{p}_1\right)\right)} $$.

In {*p*_*x*_, *p*_*y*_} representation of this energy, the following can be obtained:

$$ \hslash {\omega}_{\pm }= D\pm {M}_a\sqrt{1+2\left(1+ \cos \left({p}_x\right)+ \cos \left(\frac{3}{2}{p}_y+\frac{1}{2}{p}_x\right)+ \cos \left(\frac{3}{2}{p}_y-\frac{1}{2}{p}_x\right)\right)} $$.

After some transformations, one can obtain a standard expression (with accuracy of notations of parameters and axes) [1–5]: $\varDelta {E}_{\pm }=\pm {M}_a\sqrt{1+4{ \cos}^2\left({p}_x/2\right)+4 \cos \left({p}_x/2\right) \cos \left(3{p}_y/2\right)}$, where *ΔE*_±_≡*ℏω*_±_ − *D*.

**Fig. 2:**
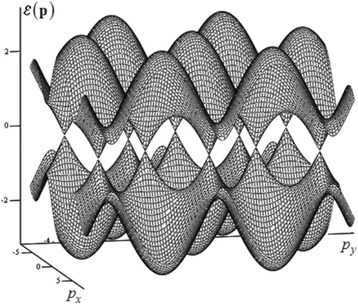
Common band structure of graphene. For each of the momenta: *p*_*x*_ and *p*_*y*_, several Brillouin zones [1] are considered, so the hexagonal structure can be seen clearly. The lower surface corresponds to the sign “–” in the formula (6) for the energy *ε*(**p**) and relates to the conduction band with normal dispersion. The upper surface corresponds to the sign “+” in this formula and relates to the conduction band with anomalous dispersion. Both zones take part in the conductivity

**Fig. 3:**
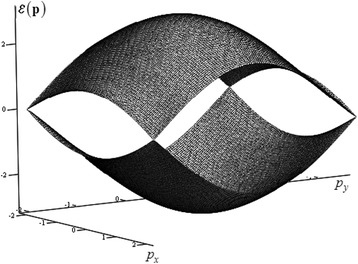
The band structure of graphene within the single-valuedness area. This area fully belong to the first Brillouin zone and it defined by the inequalities: {|*p*_*x*_|, |*p*_*y*_|} ≤ 2*π*/3

Further will be considered the dimensionless representation of this energy:

6 $$ \varepsilon \left(\mathbf{p}\right)=\pm \sqrt{1+4{ \cos}^2\left({p}_x/2\right)+4 \cos \left({p}_x/2\right) \cos \left(3{p}_y/2\right)} $$

which has the physical meaning of the dimensionless Hamiltonian for the injected electron, considered as a quasiparticle. In the Fig. 2, this energy, as it is often done, is shown within several Brillouin zones. To analyze the basic dynamical properties of quasiparticles in crystals, the consideration of the first Brillouin zone is enough. In the Fig. 3, the energy (6) is shown within the area of single valuedness, which either does not extend beyond the first Brillouin zone or coincides with it. The next subsection will analyze some features of the dynamic characteristics of the electron, as a quasiparticle in graphene conduction band within the area shown in the Fig. 3.

### Some Features of the Electron Dynamic Properties in Graphene

At first glance, the dispersion relation (6) for the electron in graphene is not very different from the same relation for simple (one atom per the cell) rectangular lattice [24–27]. In fact, it complicates the situation so that it is not always possible to carry out a full analysis of the dynamic properties of electron in graphene. Further, the two cases of the electron dynamic properties in graphene will be discussed.

#### 1. Common case

Here, both momenta, *p*_*x*_ and *p*_*y*_, are certain, nonzero and are considered, as it was already mentioned, within the area shown in Fig. 3.

One of the main dynamic characteristics of each quasiparticle is speed, as it determines the current. And it determines the electrophysical properties of graphene. Since the energy (6) has the physical meaning of the dimensionless Hamiltonian, the dimensionless speed components determined by the following relations: ${\beta}_x={\displaystyle \frac{\partial \varepsilon \left(\mathbf{p}\right)}{\partial {p}_x}}$; ${\beta}_y={\displaystyle \frac{\partial \varepsilon \left(\mathbf{p}\right)}{\partial {p}_y}}$. Substituting here the energy (6), after some transformations, the following can be obtained:

7 $$ {\beta}_x=\mp \frac{\left[2 \cos \left({p}_x/2\right)+ \cos \left(3{p}_y/2\right)\right]}{\left|\varepsilon \left(\mathbf{p}\right)\right|} \sin \left({p}_x/2\right);\kern1em {\beta}_y=\mp \frac{3 \cos \left({p}_x/2\right)}{\left|\varepsilon \left(\mathbf{p}\right)\right|} \sin \left(3{p}_y/2\right) $$

The upper (lower) signs in (7) correspond to the upper (lower) signs in the definition (6), and $\left|\varepsilon \left(\mathbf{p}\right)\right|\equiv \sqrt{1+4{ \cos}^2\left({p}_x/2\right)+4 \cos \left({p}_x/2\right) \cos \left(3{p}_y/2\right)}$. Analogous to energy (6), the lower signs correspond to the conductivity band with normal dispersion, and the upper signs correspond to conductivity band with anomalous dispersion.

If we were considering a simple crystal (one atom per the cell) with a rectangular unit cell, the components (7) would have the form:

*β*_*x*_ = sin(*p*_*x*_/2), *β*_*y*_ = sin(3*p*_*y*_/2) (8)

**Fig. 4:**
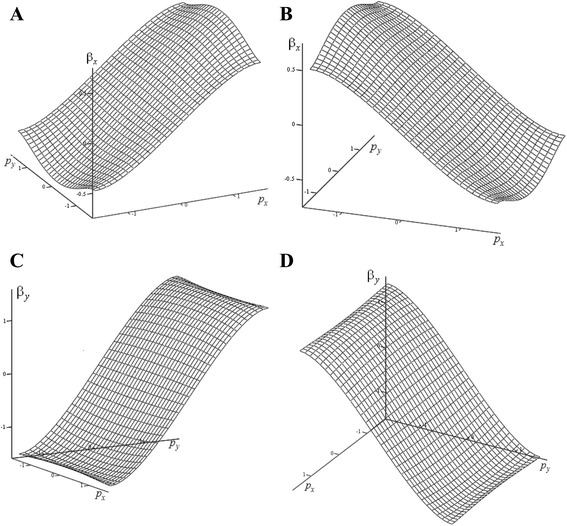
The behavior of the dimensionless velocities in the single-valuedness area. The dependence of the dimensionless velocity components *β*_*x*_, *β*_*y*_ from the dimensionless components *p*_*x*_, *p*_*y*_ of wave momentum in the single-valuedness area {|*p*_*x*_|, |*p*_*y*_|} ≤ *π*/2 presented. As it can be seen, this area is a subset of the area: {|*p*_*x*_|, |*p*_*y*_|} ≤ 2*π*/3. **a** The normal mode for *β*_*x*_ component (corresponds to the lower sign in the left formula of definitions (7)). **b** The abnormal mode for *β*_*x*_ component (corresponds to the upper sign in the left formula of definitions (7)). **c** The normal mode for *β*_*y*_ component (corresponds to the lower sign in the right formula of definitions (7)). **d** The abnormal mode for *β*_*y*_ component (corresponds to the upper sign in the right formula of definitions (7))

I.e., the analytical expression for each speed component becomes much more complicated and depends not only on its “own” component of the wave momentum. That is why, there appears the interest to numerical-graphical analysis for these components. Such analysis is important to ascertainment the question of the nature of change “behavior” of the speed components, depending on the components of the wave momentum. This analysis is shown in Fig. 4.

This figure shows both components of the dimensionless speed, defined in the formulas (7): both for the conduction band with normal dispersion (Fig. 4a, c) and for conduction band with abnormal dispersion (Fig. 4b, d). Fig. 4a, b shows both modes of *β*_*x*_ speeds (normal and abnormal). It is seen that for analyzed range, the *β*_*x*_ component practically does not depend on the component *p*_*y*_ of wave momentum; a normal speed (Fig. 4a), as it should be, increases with the increasing of component *p*_*x*_ of wave momentum; and abnormal speed (Fig. 4b) is decreased.

A similar behavior is demonstrated by both *β*_*y*_ components, (Fig. 4c, d). The only difference is that they (on the contrary) are practically independent of the momentum component *p*_*x*_. And depends on *p*_*y*_ component in a conventional manner: component *β*_*y*_ of normal speed increases with increasing of the *p*_*y*_ component (Fig. 4c), but for abnormal speed is decreased (Fig. 4d).

These numerical and graphical results are interesting because in the area {|*p*_*x*_|, |*p*_*y*_|} ≤ *π*/3_,_ these relations are simplified almost to the form (8). Since in many applications such an area is enough, then in this case, the whole dynamics of a simple rectangular lattice, which was studied in details in papers [24–27], is reproduced. The region {|*p*_*x*_|, |*p*_*y*_|} ≤ *π*/3, usually meets research needs with a good margin at the low-energy electron injection into the conduction band of graphene and when using fields of not high intensity (much lower of electrostatic threshold of material).

The other, no less important characteristic of the dynamical properties of quasiparticle, is the tensor of reciprocal effective mass. Due to the generality of consideration, this tensor is a dynamic parameter in the meaning that it depends on the components of wave momentum **p**. In accordance with the general definition: ${\mu}_{i j}^{-1}={\displaystyle \frac{\partial {\beta}_i}{\partial {p}_j}}$, the following can be obtained:

$$ {\mu}_{x x}^{-1}=\mp \frac{\left[ \cos \left({p}_x\right)+\frac{1}{2} \cos \left(3{p}_y/2\right) \cos \left({p}_x/2\right)\right]}{\left|\varepsilon \left(\mathbf{p}\right)\right|}\mp \frac{{\left[ \sin \left({p}_x\right)+ \cos \left(3{p}_y/2\right) \sin \left({p}_x/2\right)\right]}^2}{{\left|\varepsilon \left(\mathbf{p}\right)\right|}^3} $$;

$$ {\mu}_{y y}^{-1}=\mp \frac{9}{2}\frac{ \cos \left({p}_x/2\right) \cos \left(3{p}_y/2\right)}{\left|\varepsilon \left(\mathbf{p}\right)\right|}\mp \frac{{\left[3 \cos \left({p}_x/2\right) \sin \left(3{p}_y/2\right)\right]}^2}{{\left|\varepsilon \left(\mathbf{p}\right)\right|}^3} $$;

8 $$ \begin{array}{c}{\mu}_{x y}^{-1}={\mu}_{y x}^{-1}=\pm \frac{3}{2}\frac{ \sin \left(3{p}_y/2\right) \sin \left({p}_x/2\right)}{\left|\varepsilon \left(\mathbf{p}\right)\right|}\mp \\ {}\mp \left( \sin \left({p}_x\right)+ \cos \left(3{p}_y/2\right) \sin \left({p}_x/2\right)\right)\frac{3 \cos \left({p}_x/2\right) \sin \left(3{p}_y/2\right)}{{\left|\varepsilon \left(\mathbf{p}\right)\right|}^3}\kern0.5em \end{array} $$

By these two dynamic characteristics, the velocity vector and tensor of reciprocal effective mass are practically limiting the possibility of determining the dynamic parameters in general case (without limitation on the value of the wave momentum **p**). This is due to the fact that for the construction of other dynamic parameters, it is required to find the inverse transformations for Eq. (7). That is, it is necessary to determine the components of wave momentum **p**, as a function of the speed components **β** that is analytically impossible.

Therefore here, we mention one more dynamic characteristic, which can be represented explicitly. It is a wave Lagrangian. Following to the general definition of the Lagrangian: $l\left(\boldsymbol{\upbeta} \right)={\displaystyle \sum_i{\beta}_i{p}_i}- h\left(\mathbf{p}\right)$, taking into account that here *h*(**p**)≡*ε*(**p**), as well as taking into accounts the determination of the speed components (7), the Lagrangian can be obtained only in parametric form:

9 $$ \begin{array}{c} l\left(\boldsymbol{\upbeta} \right)=\frac{1}{\varepsilon \left(\mathbf{p}\right)}\left\{{p}_x\left( \sin \left({p}_x\right)+ \cos \left(3{p}_y/2\right) \sin \left({p}_x/2\right)\right)+3{p}_y \cos \left({p}_x/2\right) \sin \left(3{p}_y/2\right)-\right.\\ {}\left.-1-4{ \cos}^2\left({p}_x/2\right)-4 \cos \left({p}_x/2\right) \cos \left(3{p}_y/2\right)\right\}\kern0.5em \end{array} $$

where the parameters are the momentum components *p*_*x*_, *p*_*y*_ on which the speed components *β*_*x*_, *β*_*y*_ are dependent, as it is defined in the (7). Definition (9) in conjunction with the definitions (7) represents the parametric form of the wave Lagrangian dependence on the speed components **β**. However, its detailed study requires specific consideration, as well as the reverse conversions.

#### 2. Case of the dynamics along the axis ***x*** (***p***_***y***_ = **0**)

Here, we will consider a situation that allows fully constructing the entire dynamics of the electron in the graphene conduction band. This is the case when, for example, the external field, stimulating the current in graphene, is directed along the axis *x*. That is, when *p*_*y*_ = 0.

Immediately, it should be noted that an alternative situation (when the field is directed along the axis *y* and *p*_*x*_ = 0) does not allow to fulfill one of the reverse conversions and, therefore, to construct the full dynamics to the end.

In the case when the condition *p*_*y*_ = 0 is fulfilled, the Hamiltonian takes the form:

*ε*(**p**) = ± |2 cos(*p*_*x*_/2) + 1|.

For this situation, the first Brillouin zone is now has a diapason from − 2*π* up to 2*π*. However for illustrative purposes, we will consider not the entire first Brillouin zone, but only the area, where the structure 2 cos(*p*_*x*_/2) + 1 has a positive definiteness. This area is limited to the range: |*p*_*x*_| ≤ 4*π*/3. In this case, the energy *ε*(**p**) takes more simple form for analysis:

10 $$ \varepsilon \left(\mathbf{p}\right)=\pm \left(1+2 \cos \left({p}_x/2\right)\right) $$

Next, a single non-zero speed component can be found in accordance with the definition *β*_*x*_(**p**) = ∂*ε*(**p**)/∂*p*_*x*_:

11 $$ {\beta}_x\left(\mathbf{p}\right)=\mp \sin \left({p}_x/2\right) $$

Hereinafter, the upper (lower) signs always correspond to the upper (lower) sign in the energy (10). At the same time, we recall that the lower signs always correspond to the conduction band with normal dispersion.

An important feature of this case is that here one can find the explicit form of the analytic dependence of momentum *p*_*x*_ from speed *β*_*x*_, what was unsuccessful during general consideration. This dependence is reduced to the equation:

12 $$ {p}_x\left(\boldsymbol{\upbeta} \right)=\mp 2 \arcsin \left({\beta}_x\right) $$

and limits the area of the wave momentum *p*_*x*_ definition with the area of the function sin(…) single valuedness, i.e., with inequality |*p*_*x*_| ≤ *π*. As it can be seen, this area is a subset of the area: |*p*_*x*_| ≤ 4*π*/3.

The presence of the reverse conversion (12) immediately allows constructing the wave Lagrangian for an electron, as quasiparticle, injected into the conduction band. In accordance with the general definition: $l\left(\boldsymbol{\upbeta} \right)={\displaystyle \sum_i{\beta}_i{p}_i}- h\left(\mathbf{p}\right)\equiv {\beta}_x{p}_x-\varepsilon \left(\mathbf{p}\right)$, the following can be obtained:

$$ l\left(\boldsymbol{\upbeta} \right)=\mp \left\{2{\beta}_x \arcsin \left({\beta}_x\right)+2\sqrt{1-{\beta}_x^2}+1\right\} $$,

where it was taken into consideration that, according to (11):

13 $$ \cos \left({p}_x/2\right)\equiv \sqrt{1-{ \sin}^2\left({p}_x/2\right)}=\sqrt{1-{\beta}_x^2} $$

It is obvious that direct differentiation of this Lagrangian with respect to *β*_*x*_ gives the momentum (12), and the Lagrangian itself, as it is shown in [25, 26], is one of a phase elements of the wave function (3).

Now, we can find a single nonzero component: ${\mu}_{x x}^{-1}=\mp \frac{1}{2} \cos \left({p}_x/2\right)$. According to (13):

14 $$ {\mu}_{x x}=\mp \frac{2}{ \cos \left({p}_x/2\right)}=\mp \frac{2}{\sqrt{1-{\beta}_x^2}} $$

All values determined after energy (10) make up the so-called wave branch of the classical description of the electron conduction in graphene in the presence of motion only along the axis *x*. However, in parallel with this way of classical description, exists also a mechanical branch of description, the construction of which is based on two circumstances. First, it is based on the fact that the wave Hamiltonian (10) is also a mechanical Lagrangian. And, second, that the speed (11) is common to both descriptions. In other words, the following equality takes place: *l*_*m*_(**β**) = *ε*(**p**). Proceeding from it, as well as from the definition (11) for the speed, one can consistently find: ${l}_m\left(\boldsymbol{\upbeta} \right)=\pm \left(1+2\sqrt{1-{\beta}_x^2}\right)$.

On the basis of the definition: ${p}_m^x={\displaystyle \frac{\partial {l}_m\left(\beta \right)}{\partial {\beta}_x}}$, we derive the mechanical momentum:

15 $$ {p}_m^x=\mp 2\frac{\beta_x}{\sqrt{1-{\beta}_x^2}} $$

which exactly corresponds to the standard definition of a mechanical momentum, as the product of speed and mass. Indeed, taking into account the definition of mass (14) and in using it in definition ${p}_m^x={\mu}_{x x}\kern0.1em {\beta}_x$, we exactly obtain (15).

The last mechanical characteristic, which completes the construction of the mechanical branch of the classical type descriptions for electron conduction in grapheme, is a mechanical Hamiltonian. To construct it, we use a common definition of the Hamiltonian: ${h}_m\left({\mathbf{p}}_m\right)={\displaystyle \sum_i{\beta}_i{p}_m^i}-{l}_m\left(\boldsymbol{\upbeta} \right)\equiv {\beta}_x{p}_m^x-{l}_m\left(\boldsymbol{\upbeta} \right)$, as well as the representation inverse to (15): ${\beta}_x={\displaystyle \mp \frac{p_m^x/2}{\sqrt{1+{\left({p}_m^x/2\right)}^2}}}$. As a result we obtain: ${h}_m\left({\mathbf{p}}_m\right)=\mp \left(1+2\sqrt{1+{\left({p}_m^x/2\right)}^2}\right)$.

### Generalized Ratio of Louis de Broglie in Graphene

The generalized ratio of Louis de Broglie establishes a relationship between the mechanical and the wave momenta, more general than linear: **p**_*m*_ = **p**, which was formulated by de Broglie. This relation is of great practical importance. It allows fulfilling a correct transition from mechanical description of classical type to the wave description of classical type, for example, for the electron as a particle or quasiparticle.

Usually, the electron dynamics outside of the crystal (free electron) is considered in terms of the mechanical description of classical type. Whereas dynamic of this electron after injection into the crystal is more convenient to consider in terms of classical type too, however, for the wave branch of descriptions. If this injection takes place at a large mechanical momenta **p**_*m*_ (in the relativistic meaning), the linear relationship for such a conversion may be not suitable. This situation is clear “visible” for the case *p*_*y*_ = 0.

Indeed, from relationship (15) taking into account (11), we can find the following: ${p}_m^x=2\mathrm{t}\mathrm{g}\left({p}_x/2\right)$. In other words, for small momenta values with a good degree of accuracy, linear relationship can be used: ${p}_m^x={p}_x$. If the mechanical momentum of the electron before the injection was big enough in the relativistic meaning $\left(\left|{p}_m^x\right|>>1\right)$, then after injection, its wave momentum *p*_*x*_ will in any case satisfy the inequality |*p*_*x*_| < *π*. It is clear that the use of the linear correlation ${p}_m^x={p}_x$ in this case instead of general relationship ${p}_m^x=2\mathrm{t}\mathrm{g}\left({p}_x/2\right)$ will lead to significant errors in predicting the electrical and physical properties of graphene.

## Conclusions

Basic principles of construction of general dynamic properties an electron injected into graphene analyzed. First of all, it is shown that graphene can be regarded by a traditional method as the crystal with complex unit cell (containing two atoms). This leads to the same results as conventional for graphene but not quite traditional the consideration of them as two nested each other and interacting crystals with simple lattices. The analysis was conducted for such excited states as the electron that injected into the conduction band. This excitation is realized in the form of two dispersion law: $\varepsilon \left(\mathbf{p}\right)=\pm \sqrt{1+4{ \cos}^2\left({p}_x/2\right)+4 \cos \left({p}_x/2\right) \cos \left(3{p}_y/2\right)}$. They correspond to the dependence the electron energy from components of the wave momentum Therefore, the construction of the dynamic properties of the electron as a point object (quasiparticle) is based on the representation about this energy as a Hamiltonian.

The dimensionless velocity of the electron **β**(**p**) and the dynamic tensor of inverse effective mass ${\widehat{\mu}}^{-1}\left(\mathbf{p}\right)$ have been found for all cases considered in this paper. However, it is shown that in the general case, when the two components of the wave momentum are different from zero to construct completely all dynamics of the electron analytically it is impossible. This is due to the inability to get the relationship **β**(**p**), i.e., make analytical transformation which is an inverse with respect to **β**(**p**).

It was determined that when *p*_*y*_ = 0, *p*_*x*_ ≠ 0, i.e., when the dynamic direction is the only *x* − direction (for example, due to the orientation of the external field) to build a full dynamics of the electron analytically, it is possible (in the inverse situation *p*_*x*_ = 0, *p*_*y*_ ≠ 0, as well in a general case this is not possible). The speed, the wave Lagrangian, the dynamic tensor of effective mass (inverse and direct), the mechanical Lagrangian, the mechanical momentum, and the mechanical Hamiltonian serially found for this case. The presence of an explicit expression for the mechanical momentum allowed for this case (*p*_*y*_ = 0, *p*_*x*_ ≠ 0) to find a generalized relation of Louis de Broglie. It makes it possible to do the correct transition from classical description of mechanical type to the similar wave description. This is important because the dynamics of the free electron outside crystal is considered in terms of the mechanical description of classical type, but in the crystal it is more convenient to consider in terms of description, too, of the classical type, but wave.

It makes it possible to do the correct transition from classical description of mechanical type to the similar wave description.
